# Beyond Antibodies: B Cells and the OPG/RANK-RANKL Pathway in Health, Non-HIV Disease and HIV-Induced Bone Loss

**DOI:** 10.3389/fimmu.2017.01851

**Published:** 2017-12-22

**Authors:** Kehmia Titanji

**Affiliations:** ^1^Division of Endocrinology, Metabolism and Lipids, Department of Medicine, Emory University School of Medicine, Atlanta, GA, United States

**Keywords:** B cells, HIV, bone loss, comorbidities, cytokines, end-organ damage

## Abstract

HIV infection leads to severe B cell dysfunction, which manifests as impaired humoral immune response to infection and vaccinations and is not completely reversed by otherwise effective antiretroviral therapy (ART). Despite its inability to correct HIV-induced B cell dysfunction, ART has led to significantly increased lifespans in people living with HIV/AIDS. This has in turn led to escalating prevalence of non-AIDS complications in aging HIV-infected individuals, including malignancies, cardiovascular disease, bone disease, and other end-organ damage. These complications, typically associated with aging, are a significant cause of morbidity and mortality and occur significantly earlier in HIV-infected individuals. Understanding the pathophysiology of these comorbidities and delineating clinical management strategies and potential cures is gaining in importance. Bone loss and osteoporosis, which lead to increase in fragility fracture prevalence, have in recent years emerged as important non-AIDS comorbidities in patients with chronic HIV infection. Interestingly, ART exacerbates bone loss, particularly within the first couple of years following initiation. The mechanisms underlying HIV-induced bone loss are multifactorial and complicated by the fact that HIV infection is linked to multiple risk factors for osteoporosis and fracture, but a very interesting role for B cells in HIV-induced bone loss has recently emerged. Although best known for their important antibody-producing capabilities, B cells also produce two cytokines critical for bone metabolism: the key osteoclastogenic cytokine receptor activator of NF-κB ligand (RANKL) and its physiological inhibitor osteoprotegerin (OPG). Dysregulated B cell production of OPG and RANKL was shown to be a major contributor to increased bone loss and fracture risk in animal models and HIV-infected humans. This review will summarize our current knowledge of the role of the OPG/RANK–RANKL pathway in B cells in health and disease, and the contribution of B cells to HIV-induced bone loss. Data from mouse studies indicate that RANKL and OPG may also play a role in B cell function and the implications of these findings for human B cell biology, as well as therapeutic strategies targeting the OPG/RANK–RANKL pathway, will be discussed.

## Introduction

Rising incidences of bone loss in the form of low bone mineral density (BMD), osteopenia, and osteoporosis, and the resulting increased risk of fracture have over the past decade emerged as important non-AIDS comorbidities affecting HIV-infected individuals (1–6). Successful antiretroviral therapy (ART) over the past couple of decades has been instrumental in significantly extending the life expectancies of HIV-infected individuals to levels comparable to those of the general population (7). A significant proportion of people currently living with HIV in Europe and North America are over the age of 50 (8–10), and it is estimated that by 2030 as many as >70% of HIV-positive individuals will fall within this demographic. Similar to cardiovascular, liver and chronic kidney disease, and other comorbidities, bone loss occurs earlier and at a higher prevalence in HIV-positive individuals than in the HIV negative population (1, 8, 11). This raises concerns of a potential impending epidemic of fragility fractures and other age-associated comorbidities in this population (8, 12).

The underlying mechanisms of HIV-associated bone loss are multifactorial, given that most of the traditional risk factors for bone loss including low body mass index (BMI), older age, tobacco use, metabolic diseases, alcohol, and substance abuse are more prevalent in the HIV-infected population (10, 13). HIV infection is now however clearly established as one of the independent risk factors for bone loss (11, 14, 15), driven by the prevalence of HIV-associated risk factors including chronic inflammation, co-infection with hepatitis B or C, and paradoxically, ART (8, 10, 13). More recently, osteoimmunology has revealed the prominent role the immune system plays in bone metabolism (16) and consequently revealed that HIV-induced immune dysfunction is one of the most important contributors to bone loss.

Osteoimmunology, a term originally coined to describe studies involving the interface between the immune and skeletal systems (17), has been instrumental in our understanding of the numerous ways both organ systems are intertwined. It is now known that in various inflammatory pathological conditions characterized by bone loss, including periodontal disease (PD) and rheumatoid arthritis (RA), both cellular and soluble immune effectors can contribute to bone loss (18, 19). T cells are major contributors to bone loss in RA (20) and PD (21, 22) but their role in HIV-induced bone loss has not been elucidated. Emerging evidence now shows that B cells play an important role in bone biology in health and disease (23–25) and HIV-induced B cell dysfunction significantly contributes to HIV-induced bone loss (26).

Bone homeostasis, which is essential for maintaining skeletal integrity and strength, is regulated by a balance of bone formation by osteoblasts and resorption by osteoclasts and disruption of this balance results in bone disease (18, 27, 28). Osteoclasts are generated in a process known as osteoclastogenesis, which is driven by the key osteoclastogenic cytokine receptor activator of NF-κB ligand (RANKL). Osteoclasts originate from cells of the myeloid lineage, which in the presence of M-CSF and RANKL differentiate into receptor activator of NF-κB (RANK)-expressing pre-osteoclasts which proliferate and fuse to form giant multinucleated osteoclasts capable of resorbing bone (15, 29).

**Figure 1:**
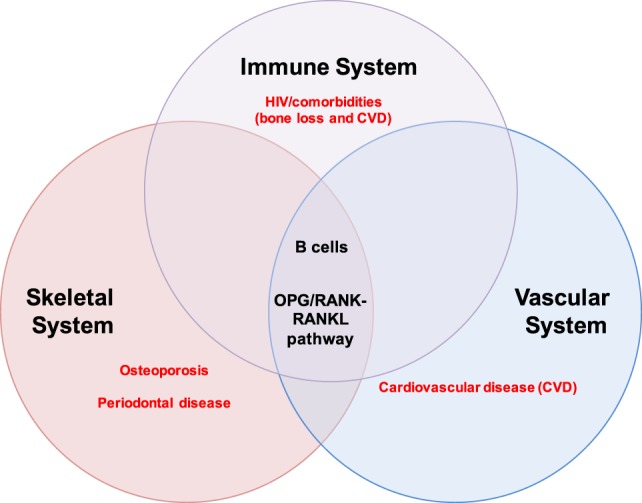
B cells and the OPG/RANK-RANKL pathway at the intersection of the immune, skeletal, and vascular organ systems. B cells mediate biological processes in health and disease *via* the OPG/RANK–RANKL pathway in three major organ systems in humans: the immune, skeletal, and vascular systems. The extensive intertwining of the immune and skeletal systems has given rise to a whole new field of study called osteoimmunology; some major pathologies implicating B cells and the OPG/RANK–RANKL pathway are highlighted in red and include osteoporosis and periodontal disease in the skeletal system, cardiovascular disease (CVD) in the vascular system, and HIV/comorbidities (bone loss and CVD) in the immune system.

Excessive osteoclast activity, as occurs in osteoporosis, results in loss of bone mass and increased susceptibility to fracture (12, 28). The effects of B and T cells on bone are mediated by several key cytokine regulators of bone metabolism (11, 18), including the inflammatory cytokines tumor necrosis factor-α (TNF-α) and interferon-γ, which have been implicated in bone loss in RA, periodontitis, postmenopausal osteoporosis, and HIV (30). Most importantly, RANKL and OPG (18) play important roles in both organ systems and perfectly illustrate the intersection of bone biology and immunity. The OPG/RANK–RANKL pathway also mediates physiological processes in the vascular system, thus intersecting with the skeletal and immune system at this axis (Figure 1).

This review will summarize our current knowledge of the role of the OPG/RANK–RANKL pathway in B cells in health and disease, and the contribution of B cells to HIV-induced bone loss. Data from mouse studies indicate that RANKL and OPG may also play a role in B cell function and the implications of these findings for human B cell biology as well as therapeutic strategies targeting the OPG/RANK–RANKL pathway will be discussed.

## The OPG/RANK–RANKL Pathway and B Cells in Health

B cells are inextricably linked to bone, from their development in the bone marrow to the homing of terminally differentiated plasma cells back to the bone marrow (30, 31) and the bidirectional regulation of the skeletal system by B cells (23, 30, 32). Osteoblasts and bone marrow stromal cells regulate B lymphopoiesis through the production of IL-7, a critical cytokine for the differentiation of early-stage B cells in the bone marrow (33, 34). Another major interaction between the skeletal system and B cells revolves around the OPG/RANK–RANKL pathway.

### B Cells and Osteoprotegerin (OPG)

The identification and characterization of OPG as a humoral regulator of bone resorption 20 years ago (35, 36) represents a major turning point in our understanding of the physiology of bone homeostasis (37, 38). OPG, named for its ability to protect bone by inhibiting osteoclast differentiation and activity, is a tumor necrosis factor receptor (TNFR) superfamily member which lacks transmembrane-spanning sequences and is secreted as a soluble protein (35, 36). OPG is the natural circulating inhibitor/decoy receptor of RANKL and can inhibit osteoclastogenesis by binding to RANKL, thus preventing bone resorption (35, 37). OPG mRNA is expressed by various tissues, including bone, brain, lung, heart, and kidney (35, 36). In the immune system, OPG is expressed in lymph nodes, B cells, and dendritic cells (DCs) and ligation of CD40 upregulates its expression (39).

Osteoblasts and their precursors were previously considered to be the primary source of OPG in the bone marrow (40, 41) but B lineage cells are now known to account for over 60% of total bone marrow OPG production (25). B cell knockout (KO) mice were osteoporotic and deficient in bone marrow OPG, confirming the critical role of B cells in the preservation of bone homeostasis and attainment of peak bone mass (25).

Unlike its role in bone homeostasis, the role of OPG in B cell function is less well documented. OPG KO mice develop severe osteoporosis due to unchecked osteoclastogenesis and bone resorption (42, 43). Interestingly, OPG-deficient mice also accumulated transitional/immature B cells in their spleens, and generated impaired antibody (Ab) responses to a T cell-dependent (DNP-KLH) antigen (Ag) challenge, suggesting that OPG may regulate B cell maturation and development of efficient Ab responses (44).

### B Cells and RANKL

The ligand for OPG is identical to a TNFR family member called TNF-related activation-induced cytokine or RANKL (37, 45). Human RANKL exists in two forms: a cellular, membrane-bound form and a soluble form, and both forms were shown to be biologically capable of promoting osteoclast formation (46, 47). RANKL is also expressed in a variety of tissues, including bone marrow and lymphoid tissues (36, 47, 48). RANKL is best known for its indispensable role in the complete differentiation of mature osteoclasts (36, 37, 47). Unlike OPG, resting B cells have not been conclusively shown to produce significant amounts of RANKL, but activated B cells are an important source (23), particularly in inflammatory disease states.

### B Cells and RANK

The receptor for RANKL, RANK, was initially identified on DCs (48) and later discovered to be expressed on preosteoclastic cells (37, 46, 49, 50) and B cells (39, 51). The binding of RANKL to RANK stimulates osteoclastogenesis, resulting in bone-resorbing osteoclasts (47).

Lack of functional RANK in both humans and mice results in osteopetrosis due to the absence of osteoclasts (19, 49, 52). Mice deficient in RANK had defects in B cell development which resulted in reduced numbers of mature B cells in the periphery (49). Humans with mutations in RANK also had B cell defects including hypogammaglobulinemia and impaired Ag-specific Ab responses (52).

## The OPG/RANK–RANKL Pathway and B Cells in Non-HIV Disease

Osteoprotegerin, RANK, and RANKL are produced by a wide variety of cells and tissues in three major organ systems: the vascular, immune, and skeletal systems and are thus implicated in the pathogenesis of various diseases in these organs (15, 38) (Figure 1). Although best known for its involvement in the pathogenesis of osteoporosis and other bone diseases such as Paget’s disease of bone (53–55) and PD (38, 56), the OPG/RANK–RANKL pathway has also been implicated in other diseases including RA (14, 38, 57) and CVD (58–60).

### Rheumatoid Arthritis

The bone and joint destruction that occurs in the autoimmune disorder RA results from increased RANKL-induced osteoclastic bone resorption in the synovial joints (57, 61, 62). Several immune cells have been identified as the sources of RANKL in the arthritic synovium, including Th17 cells (63), macrophages, DCs (57), and activated B cells (64). Targeted B cell depletion therapy for RA using the anti-CD20 Ab rituximab suggests that B cells play a critical role in RA-associated joint damage (64–66). B cells were shown to contribute to RA pathogenesis through their Ag-presenting function, autoantibody production, and cytokine secretion (66, 67). A link between B cells and joint destruction in RA has been confirmed by studies demonstrating that Rituximab significantly reduces RANKL levels in the synovium (68, 69). This link has recently been confirmed by studies identifying pro-inflammatory B cells as major sources of RANKL in RA (64, 66). These findings highlight the importance of Ab-independent (cytokine-producing) B cell functions in the pathogenesis of disease and make a case for the therapeutic potential of targeting the B cell OPG/RANK–RANKL pathway in RA and other diseases.

In contrast to RANKL, multiple studies have demonstrated that serum levels of OPG are elevated in RA, resulting in a decreased RANKL/OPG ratio (70, 71). Elevated OPG levels were independently associated with RA disease severity and CVD, and it has been suggested that OPG concentration could be used as a predictive marker for assessing RA-associated CVD risk (72, 73). Data on the role of B cell-produced OPG in the pathophysiology of RA are however lacking.

### Cardiovascular Disease

A role for the OPG/RANK–RANKL pathway in the pathogenesis of vascular calcification and CVDs has been established for over a decade now. Both OPG and RANKL have been detected in atherosclerotic plaques (74) and an increased RANKL/OPG ratio is associated with atherosclerosis (59). Transgenic expression of OPG in OPG KO mice prevented the development of arterial calcification but exogenous OPG administration did not reverse existing calcification, suggesting that similar to bone, OPG is a protective factor in the cardiovascular system (75, 76). Results in human studies however seem to conflict with the animal studies, with higher OPG levels consistently associated with CVD incidence (76, 77). The contribution of B cells to OPG/RANK–RANKL-linked CVD has however not been clearly elucidated. Low-density lipoprotein (LDL) receptor KO mice (LDLR^−/−^) were B cell deficient and developed atherosclerosis, suggesting that B cells and/or antibodies are protective against atherosclerosis (78); it is conceivable that OPG produced by B cells mediates this protective effect.

### Bone Diseases

#### Osteoporosis

Osteoporosis is characterized by loss of bone mass and mineral density resulting from an excess of bone resorption by osteoclasts relative to bone formation by osteoblasts (18, 27, 28). The role of the OPG/RANK–RANKL pathway in the pathogenesis of osteoporosis has been well documented and extensively reviewed (15, 37, 38, 62); the role of B cells is however still being elucidated.

Postmenopausal osteoporosis, the most common form of osteoporosis, arises from decreased estrogen levels (62) and was shown in both human patients and an animal model to be linked to increased RANKL expression by B cells (79). Mice subjected to ovariectomy, commonly used as an animal model of estrogen deficiency, have increased numbers of B cells, suggesting that B cells may play a role in estrogen-deficiency osteoporosis (79–81). Data on the contribution of B cells to ovariectomy-induced bone loss is however conflicting. Some studies have demonstrated that ovariectomy-induced bone loss occurs independently of mature B cells (82) and others show that ovariectomy-induced bone loss is linked to RANKL expression on immature B cells (79). Given the fact that B cells are able to express RANKL at various stages in their differentiation, this raises the possibility that the contribution of B lineage cells to estrogen-deficiency osteoporosis is dependent on the differentiation/maturation stage of the B cell. Beyond the differentiation stage however, the activation status of B cells seems to be a better indicator of their ability to produce bone-damaging RANKL (23). This is especially relevant in the context of inflammatory diseases like RA, PD, and HIV-induced bone loss.

#### Periodontal Disease

Periodontal diseases are inherited or acquired disorders affecting the supporting structures of the teeth and affect as many as 50–90% of the world’s population (83). The underlying microbial infections were traditionally the focus of majority of the research on the pathogenesis of PDs but in recent years the focus has shifted to the role of the host response/factors in pathogenesis (83, 84). Host immune/inflammatory responses are critical for pathogenesis and inflammation (84) and the term PD generally refers to inflammation-induced disorders, ranging from the mildest form (gingivitis) to the more invasive severe periodontitis (83). Unlike gingivitis which is completely reversible by effective regular oral hygiene, periodontitis extends deeper into the tissue and can result in the permanent loss of the supporting structures of the teeth and alveolar bone (83).

One of the microorganisms most commonly implicated in PD pathogenesis is *Actinobacillus actinomycetemcomitans (Aa)*, which induces RANKL expression on a variety of cell types infiltrating in PD lesions (84). While the RANKL levels in PD lesions are consistently elevated in most clinical studies, some studies found lower (22) or unchanged (24) OPG levels in lesions, which both resulted in higher RANKL/OPG ratios in periodontitis compared to healthy controls (22, 24, 84). Activated B and T cells were shown to be the primary source of RANKL in gingival tissues from individuals with periodontitis (24, 85). B cell percentages in chronic PD lesions were associated with disease severity, suggesting that B cells promote PD (86) and interestingly, PD lesion-infiltrating B cells in humans were activated transitional CD5^+^ cells (86, 87). Using a rat model, it was also demonstrated that B cells contributed to osteoclast formation and periodontal bone loss by secreting RANKL following activation by Aa in a T cell-independent manner (85).

## B Cells, The OPG/RANK–RANKL Pathways, and HIV-Induced Bone Loss

With the availability of ever-improving treatment regimens, ART is enabling HIV-infected individuals to live longer than ever before, but life expectancies of patients remain lower than those of the general population (7, 12, 15, 16, 59). Over 33% of people currently living with HIV in Europe are >50 years of age and this percentage is expected to increase to >70% by 2030 (88); in the US, the same demographic is estimated to constitute up to 50% of the HIV-positive population (89). This increased longevity is however accompanied by earlier occurrence and higher prevalence of several non-AIDS end-organ comorbidities including cardiovascular and bone diseases (90–92), which in turn imposes significant disease burdens on the patients, healthcare systems, and society.

As discussed above, under inflammatory conditions, B cells produce higher amounts of RANKL, leading to an increased RANKL/OPG ratio, which drives disease progression (26, 29) in inflammatory diseases such as RA. HIV infection is associated with persistent inflammation (93) and the success of B cell-targeted/depleting therapies in reducing inflammation in autoimmune disorders such as RA suggest that B cells may contribute to persistent inflammation (94, 95). Given the pivotal role this pathway plays in osteoclastogenesis and bone loss, its role in B cells and HIV is perfectly illustrated by its contribution to inflammation-driven HIV-induced bone loss.

**Figure 2:**
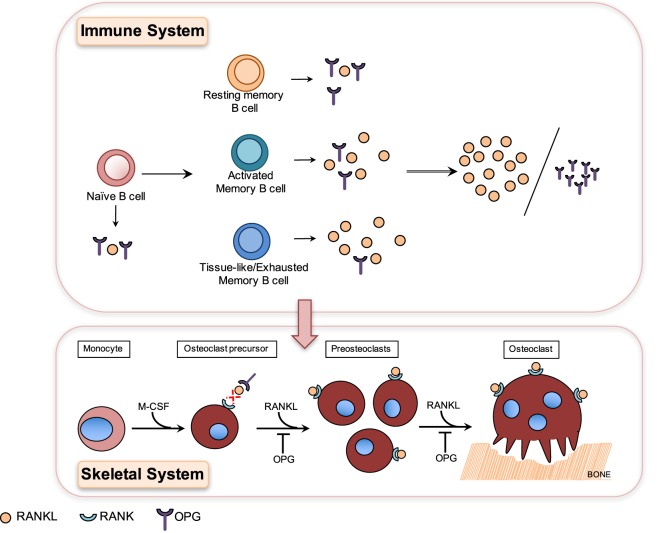
Differential production of osteoprotegerin (OPG) and receptor-activator of NF-κB ligand (RANKL) by B cell subsets results in higher RANKL/OPG ratio, which contributes to HIV-induced osteoclastogenesis and bone loss. Osteoclasts are generated in a process known as osteoclastogenesis, which is driven by the key osteoclastogenic cytokine RANKL. Osteoclasts originate from cells of the myeloid lineage, which in the presence of M-CSF and RANKL differentiate into receptor-activator of NF-κB (RANK)-expressing pre-osteoclasts, which proliferate and fuse to form giant multinucleated osteoclasts capable of resorbing bone. HIV infection leads to the depletion of resting memory B cells and expansion of activated B cell subsets including activated memory and tissue-like memory B cells. Resting memory B cells produce the highest amounts of OPG and tissue-like memory B cells conversely the lowest amounts of OPG and the highest amounts of RANKL (26). HIV-induced B cell subset changes therefore translate into higher RANKL/OPG ratios, which contribute to increased osteoclastogenesis and bone loss in HIV-infected patients.

A hallmark of chronic HIV infection is the altered distribution of subsets in the B cell compartment (93), notably the loss of resting memory B cells (26) and the expansion of exhausted/tissue-like memory B cells (26, 93, 96). Interestingly, OPG expression was lowest in the HIV-expanded tissue-like memory B cell subset, which conversely showed higher RANKL expression (26) (Figure 2). This tissue-like memory B cell subset was also previously shown to express the inhibitory receptor FcRL4 (96), which in RA defined a pro-inflammatory RANKL-producing B cells subset (66). Taken together, this suggests that inflammation does drive B cell subset RANKL expression in HIV infection.

Low BMD increases the risk of fragility fractures and is widely prevalent in HIV-infected individuals, with as many as 67% presenting with osteopenia and ~15% with osteoporosis (91). Increased osteopenia and osteoporosis rates translate into significantly elevated fracture risk, and studies show that HIV-infected individuals do indeed suffer more fragility fractures, at younger ages, than the general population (1). The ubiquitous presence of traditional risk factors for low BMD such as increased smoking and low BMI in most HIV-infected cohorts complicates efforts to understand and elucidate the mechanisms underlying HIV-induced bone loss (11, 26, 97). HIV infection in itself is now recognized as a risk factor for bone loss (97).

HIV transgenic rats almost perfectly mimic the clinical hallmarks of human HIV-induced bone disease, including profound skeletal damage. Bone loss in this model was driven by increased B cell RANKL expression concurrent with decreased OPG expression, which in turn resulted in increased RANKL/OPG ratio and thus osteoclastogenesis and bone loss (98). This mechanism of HIV-induced B cell dysfunction-driven bone loss was later confirmed in a clinical study of untreated HIV-infected individuals where it was demonstrated that increased B cell RANKL/OPG was indeed associated with increased bone resorption (26). This demonstrated for the first time that the OPG/RANK–RANKL pathway is indeed a key pathway utilized by B cells to effect skeletal damage in HIV infection. This demonstrates clearly how HIV-induced B cell changes in the immune system translate directly into dysfunction and bone loss in the skeletal system (Figure 2).

## Regulatory Effects of the OPG/RANK–RANKL Pathway on B Cells and Humoral Immune Responses

Due to the expression of OPG, RANK, and RANKL on a wide variety of immune cell types, the pathway is thought to play an important role in immune cell biology. Despite the involvement of B cell-expressed OPG and RANKL in the normal function of the immune, skeletal, and vascular systems and in the pathogenesis of multiple diseases, the effect of these molecules on B cell physiology has not been extensively described.

Receptor-activator of NF-κB ligand plays an important role in the development of secondary lymphoid organs. RANK- and RANKL-deficient mice had poorly developed or completely lacked secondary lymphoid tissues including lymph nodes, Peyer’s patches, cryptopatches, and spleen (46, 49, 62).

The role of this pathway in B cell function has also been investigated in a few mouse studies. OPG-deficient mice accumulated transitional/immature B cells in their spleens and generated impaired Ab responses to a T cell-dependent (DNP-KLH) Ag challenge, suggesting that OPG may regulate B cell maturation and development of efficient Ab responses (44). Conversely, B cell development was impaired in RANKL-deficient mice, suggesting that OPG regulates B cell development.

In another study (99), OPG was used to treat mice induced to develop different types of cellular and humoral immune responses through: (1) infection with *Mycobacterium bovis* Bacillus Calmette and Guerin (BCG) followed by OPG-Fc treatment, (2) immunization with KLH in Freund’s adjuvant or by i.p. injection of a Pneumococcal Vaccine Polyvalent (Pneumovax^®^23, Merck) (3) immunization with Keyhole Limpet Hemocyanin (KLH) *in vivo* followed by OPG-Fc treatment, and (4) In a bid to induce contact hypersensitivity, mice were also sensitized with the hapten oxazolone, followed by treatment with OPG-Fc. T and B cells were also exposed to OPG *in vitro*. OPG treatment did not affect cell-mediated responses including contact hypersensitivity but increased humoral immune responses to KLH and the pneumococcal vaccine. *In vitro*, OPG modestly stimulated T cells but not the proliferation of B cells. These results demonstrated that OPG has modest regulatory effects on humoral immune responses to certain Ags. The potential impact of the OPG/RANK–RANKL on the generation of human humoral immune responses is not clear and definitely merits further study.

## Therapeutic Strategies Targeting the OPG/RANK–RANKL Pathway

Although initially described in the context of bone disease, the OPG/RANK–RANKL pathway is now known to influence normal physiology and pathology in the immune, skeletal, and vascular systems. This opens up the potential for a lot of cross application of potential therapeutic strategies targeting this pathway.

One such strategy involves RANKL inhibition; *E. coli*-derived Fc-OPG showed great promise in phase I trials, causing rapid decline in bone turnover markers in postmenopausal women (100), also serving as a proof of concept that RANKL blockade could meaningfully impact bone turnover in humans (46). Perhaps the best known RANKL inhibitor in clinical use to date is denosumab, a fully human IgG2 Ab which binds RANKL with high affinity and unlike Fc-OPG does not bind to mouse and rat RANKL and TRAIL (46). In clinical use, denosumab effectively reduces fracture risk by reducing bone resorption and was shown to be superior to bisphosphonates in its ability to increase BMD in postmenopausal women (46). When used to treat cancer-induced bone disease, denosumab effectively reduced levels of bone turnover markers in patients with solid tumor (breast, prostate, and lung) metastases to bone and prolonged bone metastasis-free survival and delayed the onset of first metastasis in certain prostate cancers (101). Denosumab was also well-tolerated and no significant changes in B cell numbers were noted (102, 103). The effect of denosumab on B cell function is not fully elucidated; in one study investigating its utility as a postmenopausal osteoporosis treatment (104), 2/412 women developed transient non-neutralizing anti-denosumab antibodies, which did not adversely affect the skeleton but did appear to alter the effectiveness of the drug (104). Due to the wide pattern of expression of RANKL, including on lymphocytes, and in the vascular and skeletal systems, RANKL inhibition using denosumab could potentially increase susceptibility to infections and neoplasias (105), particularly in immunocompromised patients. No significant alterations in inflammation and immunity have however been observed in preclinical and clinical studies of denosumab, although rare cases of severe skin infections of the lower extremities were reported (106). To date, no data are available on the use of denosumab in HIV infection and the effect of RANKL blockade on humoral immune responses in HIV-infected individuals remains to be elucidated.

## Conclusion

In summary, B cells are intricately intertwined with the OPG/RANK–RANKL pathway, plays important roles in the immune, skeletal, and vascular systems, and much remains to be discovered about the influence of this pathway on human humoral immune responses.

## Author Contributions

KT designed, drafted, and revised the manuscript and is accountable for all aspects of the manuscript.

## Conflict of Interest Statement

The author declares that the research was conducted in the absence of any commercial or financial relationships that could be construed as a potential conflict of interest.
